# Novel MicroRNAs Differentially Expressed during Aging in the Mouse Brain

**DOI:** 10.1371/journal.pone.0040028

**Published:** 2012-07-23

**Authors:** Sachi Inukai, Alexandre de Lencastre, Michael Turner, Frank Slack

**Affiliations:** Department of Molecular, Cellular and Developmental Biology, Yale University, New Haven, Connecticut, United States of America; The Roslin Institute, University of Edinburgh, United Kingdom

## Abstract

MicroRNAs (miRNAs) are endogenous small RNA molecules that regulate gene expression post-transcriptionally. Work in *Caenorhabditis elegans* has shown that specific miRNAs function in lifespan regulation and in a variety of age-associated pathways, but the roles of miRNAs in the aging of vertebrates are not well understood. We examined the expression of small RNAs in whole brains of young and old mice by deep sequencing and report here on the expression of 558 known miRNAs and identification of 41 novel miRNAs. Of these miRNAs, 75 known and 18 novel miRNAs exhibit greater than 2.0-fold expression changes. The majority of expressed miRNAs in our study decline in relative abundance in the aged brain, in agreement with trends observed in other miRNA studies in aging tissues and organisms. Target prediction analysis suggests that many of our novel aging-associated miRNAs target genes in the insulin signaling pathway, a central node of aging-associated genetic networks. These novel miRNAs may thereby regulate aging-related functions in the brain. Since many mouse miRNAs are conserved in humans, the aging-affected brain miRNAs we report here may represent novel regulatory genes that also function during aging in the human brain.

## Introduction

Aging is a complex process that manifests a variety of characteristic and evolutionarily conserved changes. In higher organisms, aging is often accompanied by cognitive decline and is also cited as the primary risk factor for many neurodegenerative disorders including Alzheimer’s disease, Parkinson’s disease, and trinucleotide repeat disorders [1]–[3]. While some physiological differences between young and aged brains have been described, such as the differential recruitment of specific brain regions and synaptic loss [4], [5], the mechanisms involved in brain aging at the molecular level are not well understood.

Gene expression profiling studies of the brain have been conducted in mice, rats, chimpanzees, and humans and have shown that specific genes and pathways are affected by aging [6]–[10]. For example, studies in mice have reported that genes involved in inflammatory and stress responses, as well as many proteases involved in neuropeptide metabolism, show altered expression [6], [9]. How the many affected pathways and processes are regulated and fit into the overall picture of brain aging remain to be elucidated.

MicroRNAs (miRNAs) are endogenous small RNA molecules that control gene expression post-transcriptionally, primarily through binding to complementary target sequences in the 3′ untranslated regions (UTRs) of mRNAs. Work in *Caenorhabditis elegans* has demonstrated that miRNAs function in aging and a variety of age-associated pathways and processes [11]–[13]. Brain-specific/−enriched miRNAs have been identified [14] and associated with brain development and neurodegeneration [15]. Furthermore, central nervous system-expressed miRNAs have been shown to demonstrate a wide variety of spatial and temporal expression patterns [16], [17]. However, neither their specific functions nor the gene regulatory networks with which they are associated have been studied in detail, and we do not understand how miRNAs are affected in the aging brain.

In this study, we used deep-sequencing technology to investigate the expression changes of miRNAs during aging in the mouse brain. Our results show that many miRNAs, including several novel miRNA candidates, are significantly differentially expressed during aging with a global downward trend of miRNA expression, in agreement with other studies of miRNA expression changes during aging [12], [18]. Furthermore, predicted targets of these age-associated miRNAs have known roles in aging processes, such as in insulin signaling. Together, our study provides further support for the proposed roles for miRNAs in aging and age-related events in the murine brain.

## Results

### Mouse Brain Small RNA Cloning and Sequencing

In order to understand potential contributions of miRNAs to aging processes in the mouse brain, we first examined their expression changes using deep sequencing with Solexa technology. cDNA libraries from small RNAs were prepared from whole brains of young and old mice (5 months and 24–25 months, respectively). A total of 14,034,295 raw sequences of small RNAs were obtained by Solexa deep sequencing (Illumina). After alignment to the mouse genome (mm9), the resulting total number of genome-aligned reads was 11,181,999 (80%). The number of sequence reads that correspond to known miRNAs – 7,049,494 (63% of genome-matching reads) – was determined by perfect sequence matching to the database of known miRNAs (miRBase release 16). We checked for the expression of 1055 different mouse miRNA types annotated in miRBase (release 16) [19] – these include star sequences as well as alternate miRNA products (denoted as −5p/−3p or, sometimes, -s and -as). We detected perfect matches to 558 of these miRNA products in our mouse brain samples (**Table S1**).

To compare the differential expression of miRNAs in the brain of young mice versus old mice, the number of miRNAs in each sample was normalized to the total number of reads (sequencing depth) in each sample that matched the mouse genome (mm9) (old/young  = 1.472107). At least 184 miRNAs were considered to be differentially expressed (P-value <0.05, as determined by DEGseq [20]) (**Table S2**). To generate a conservative list, we filtered this data set by considering only miRNAs with read counts ≥10 in at least one time point sample (miRNAs with fewer reads did not exhibit dramatic expression changes). This resulted in 172 miRNAs (bolded in **Table S2**). An additional 108 sequences were previously annotated as miRNA star sequences and are reported separately (**Table S3**). Candidates that we initially identified in our novel miRNA discovery pipeline (see below) but were subsequently reported in the latest release of miRBase (release 16) were also added to the list of differentially expressed known miRNAs.

### Novel miRNA Discovery

**Figure 1:**
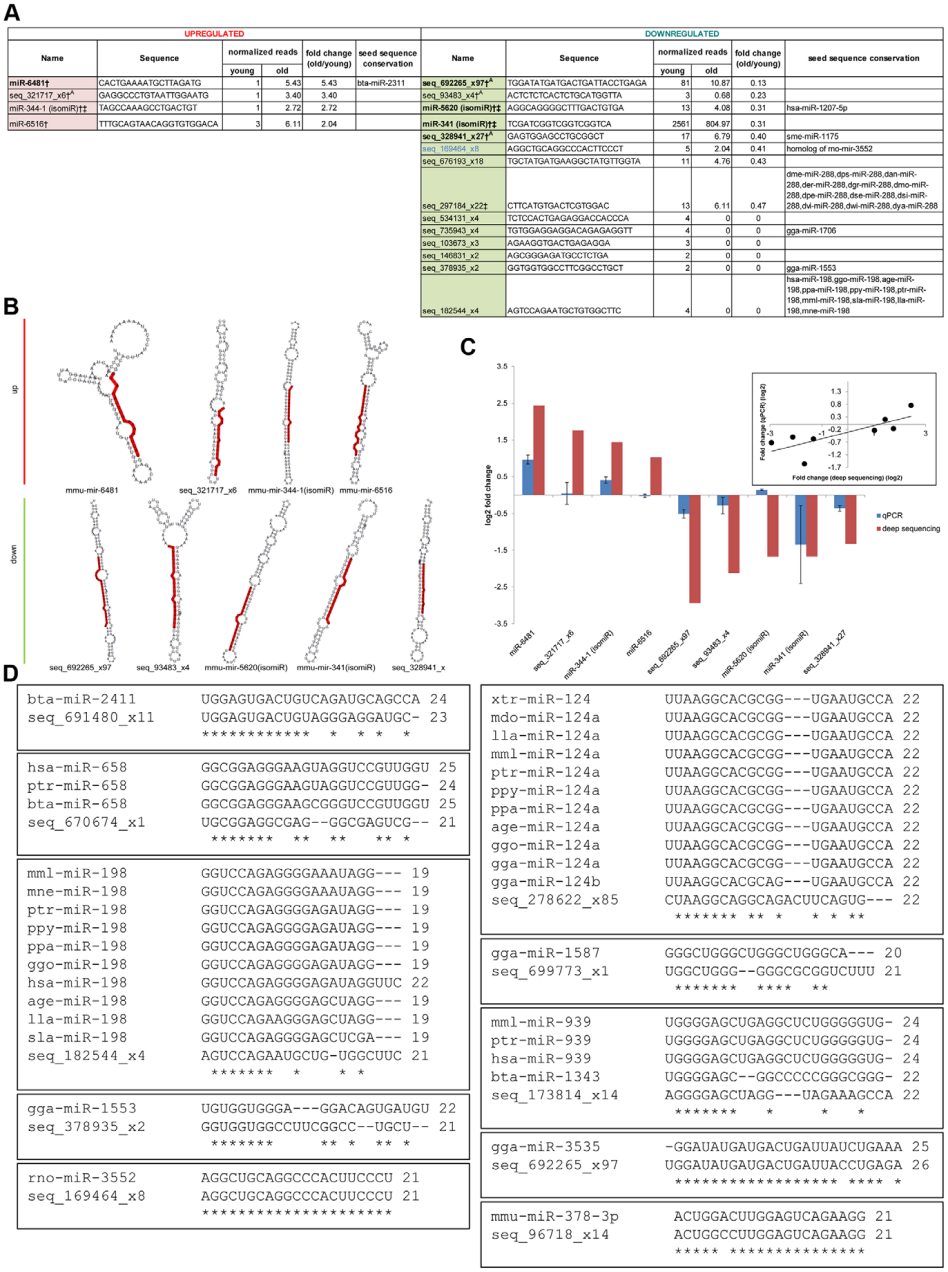
Novel miRNA candidates. (**a**) Novel miRNA candidates that change more than 2.0-fold in expression in old versus young mouse brains. MiRNA frequency was normalized by all reads that matched to the mouse genome (mm9) (Old/Young  = 1.472107). †: candidates validated by qRT-PCR. ‡: candidates with sequence overlap with known miRNAs (but have distinct mature miRNA sequences: isomiRs). ^A^: Novel miRNA candidates that map to regions overlapping snoRNA and rRNA sequences (see main text). Blue font: miRNA novel to mouse. Black font: completely novel miRNA sequence, excluding seed sequence matches. Differentially expressed miRNAs with P-values <0.05 (calculated using DEGseq [20]) indicated in bold. See also Table S4. (**b**) Secondary structures of putative precursor hairpins corresponding to nine novel miRNA candidates identified in this study. The predicted miRNA mature sequences are highlighted in red. Four of these novel miRNAs were found to be up-regulated (top) in aged mouse brain while five others were down-regulated (bottom). (See also Table S4). (**c**) Comparison of qRT-PCR data with deep sequencing data for the nine novel miRNA candidates shown in (b). Values shown are log2 ratios of old versus young brain expression levels. qPCR results were normalized to U6 snRNA expression levels. Inset: Correlation of expression changes as measured by deep sequencing versus qPCR (Pearson correlation coefficient  = 0.78). Plot for miR-5620 (isomiR) was taken out as the sequence was not reliably detected by qPCR. (**d**) Sequence alignment of novel miRNA candidates with known miRNAs of other species. *: conserved nucleotide. age: *Ateles geoffroyi*. bta: *Bos taurus*. dan: *Drosophila ananassae*. der: *Drosophila erecta*. dgr: *Drosophila grimshawi*. dme: *Drosophila melanogaster*. dmo: *Drosophila mojavensis*. dpe: *Drosophila persimilis*. dps: *Drosophila pseudoobscura*. dse: *Drosophila sechellia*. dsi: *Drosophila simulans*. dvi: *Drosophila virilis*. dwi: *Drosophila willistoni*. dya: *Drosophila yakuba*. gga: *Gallus gallus*. ggo: *Gorilla gorilla*. hsa: *Homo sapiens*. lla: *Lagothrix lagotricha*. mdo: *Monodelphis domestica*. mml: *Macaca mulatta*. mne: *Macaca nemestrina*. ppa: *Pan paniscus*. ppy: *Pongo pygmaeus*. ptr: *Pan troglodytes*. rno: *Rattus norvegicus*. sla: *Saguinus labiatus*. sme: *Schmidtea mediterranea.* xtr: *Xenopus tropicalis*.

To uncover novel miRNAs, we used the software miRDeep, which was developed to identify novel miRNAs in deep sequencing data [21]. The algorithm takes into account several factors to score the probability that a candidate small RNA found by deep sequencing represents a *bona fide* miRNA: the presence in the deep sequencing data of reads corresponding to typical products of miRNA biogenesis; the stability of the putative pre-miRNA hairpin; and homology to previously identified miRNAs. Beginning with 11,181,999 genome-matching reads, and after discarding reads corresponding to previously annotated regions, miRDeep analysis revealed the existence of a total of 41 novel candidate miRNAs (**Figure 1(a)** **, Table S4**).

Some of these novel miRNA candidates share seed sequences with known miRNAs in mouse and other species (**Figure 1(a)** **, (d)**), as determined by the miRDeep algorithm or through BLAST sequence alignment, providing additional evidence that they are *bona fide* miRNAs. Interestingly, the mature sequence of one novel miRNA candidate, seq_96718_x14, is nearly identical to miR-378-3p (one nucleotide difference), but alignment of the precursor sequence mapped seq_96718_x14 to chromosome 10 rather than to chromosome 18 on which miR-378 resides. These precursor sequences share little conservation. Thus, while seq_96718_x14 may be a *mir-378* family member, it is distinct from miR-378-3p and not merely a single nucleotide variant occurring from the same locus. Indeed the differing nucleotide falls within the seed region (**Figure 1(d)**), suggesting that the two miRNAs regulate different sets of genes. One miRNA matched perfectly with a miRNA in another species, suggesting it to be a mouse homolog. Novel miRNA candidate seq_169464_x8 aligned perfectly with rno-miR-3552; the genomic contexts of the two miRNAs are also conserved. It is important to note that some novel miRNA candidates were mapped to genomic regions overlapping known miRNA gene sequences, but the mature sequences were distinct and therefore considered distinguishable from the overlapping known miRNAs. These miRNAs are referred to as alternative forms (isomiRs) of the known miRNAs in this paper (miR-341 isomiR, miR-344-1 isomiR, and miR-5620 isomiR). Other miRNA candidates did not share sequences with any known miRNAs and may represent a group of miRNAs expressed only in aging- or brain-specific contexts. Three novel miRNA candidates overlapped with small nucleolar RNA (snoRNA) sequences (miR-6516, seq_692265_x97, and seq_93483_x4), and two more mapped to several genomic loci when BLASTed including ribosomal RNA (rRNA) sequences (seq_328941_x27 and seq_321717_x6).

### Validation of Candidate Novel miRNAs

We next evaluated the likelihood that novel miRNA candidates represent true miRNAs. The secondary structures of putative pre-miRNA hairpins were generated using RNAfold [22]; structures of select novel miRNA candidate precursor sequences are shown in **Figure 1(b)**. Although demonstrating differing complexities, all novel miRNA candidate precursor sequences fold into hairpin structures characteristic of miRNA precursors. With respect to genomic locations, novel miRNA candidates are found in exonic, intronic, and intergenic regions, with no apparent bias. Finally, we tested the expression of nine novel miRNA candidates via TaqMan qRT-PCR and confirmed the expression of 8 (**Figure 1(c)**). miR-5620 (isomiR) was detected but with late C_p_ call.

### Mapping Novel miRNAs to Genomic Locations

**Figure 2:**
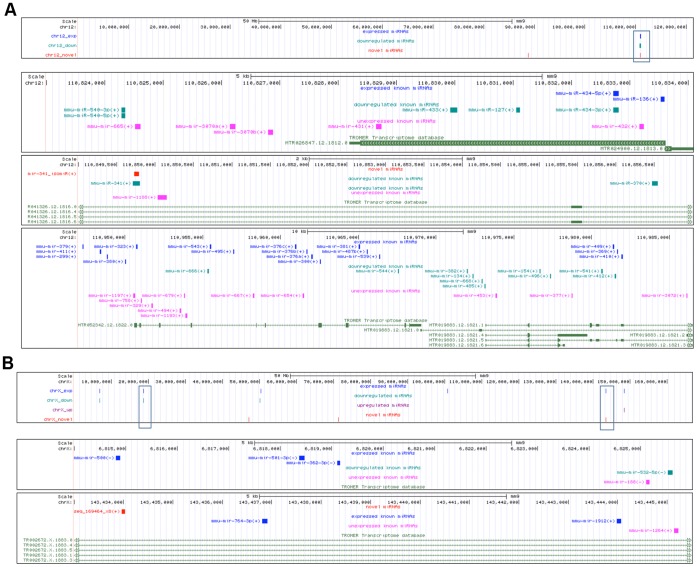
Genomic locations of brain-expressed miRNAs found within 10 kb region of each other. (a) miRNA clusters found on chromosome 12. Top panel: location of miRNAs relative to the entire chromosome 12. Bottom three panels: three distinct clusters found in the boxed off region in the top panel. The three clusters are located close to each other. (b) miRNA clusters found on X chromosome. Top panel: location of miRNAs relative to the entire X chromosome. Bottom two panels: clusters found in the boxed off regions of the top panel, from left to right. Blue: brain-expressed known miRNAs. Teal: miRNAs downregulated in expression in our dataset. Purple: miRNAs upregulated in expression in our dataset. Pink: known miRNAs found in the same genomic region but not found to be expressed in our dataset. Red: brain-expressed novel miRNA candidates. Green: TROMER transcriptome data (retrieved from UCSC Genome Browser).

We examined the genomic locations of brain-expressed miRNAs to see if miRNAs found in particular genomic regions were potentially coexpressed and, therefore, potentially coregulated. Clusters of known miRNAs were retrieved from the miRBase website. Many miRNAs are located within close proximity (<10 kb) on chromosomes 12 and X suggesting that these miRNAs might be coexpressed (**Figure 2**). Several novel miRNA candidates also were found in close proximity to these clusters. Some of these miRNAs mapped to the same transcript. It may be important to note, however, that not all known miRNAs lying within 10 kb of brain-expressed miRNAs were expressed in our data sets, thereby suggesting distinct expression regulatory mechanisms for these miRNAs.

### Dynamic Expression of miRNAs in Brain Aging

**Figure 3:**
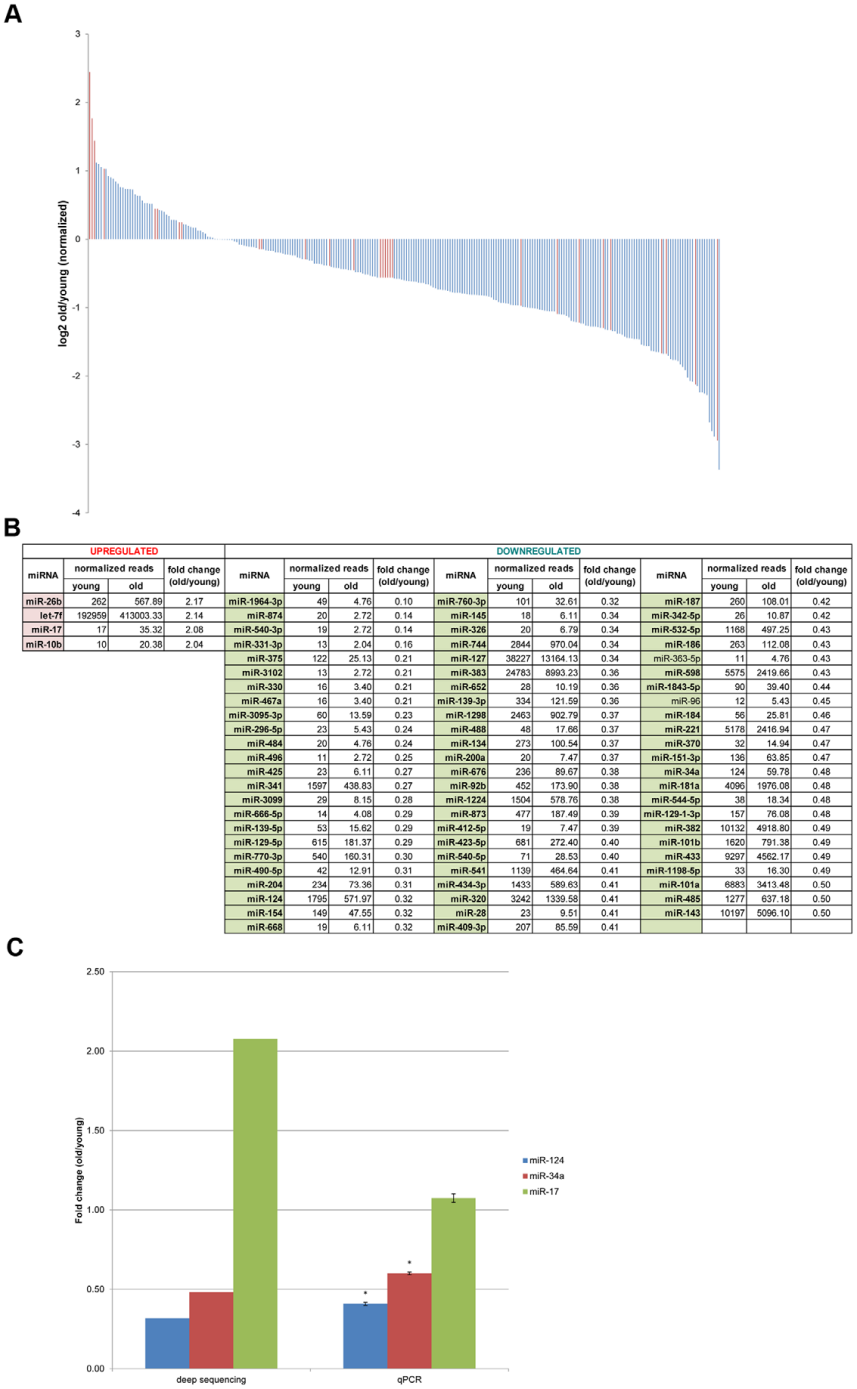
Brain-expressed miRNAs. (**a**) Expression changes of miRNAs in mouse brain with aging. Distribution of individual miRNA expression changes are ranked by those miRNAs that exhibit the greatest increase in expression with aging (log2 Ratio (Old/Young)). Blue: known miRNAs, red: novel miRNAs. (**b**) Known miRNAs that change more than 2.0-fold in expression in old versus young mouse brains. Only miRNAs with at least 10 sequence reads at one time point are shown and P-value <0.05 are in bold. MiRNA frequency was normalized by all reads that matched to the mouse genome (mm9) (Old/Young  = 1.472107). (**c**) Comparison of qRT-PCR data with deep sequencing data for three known miRNAs. Values shown are fold changes in old versus young brain expression levels. qPCR results were normalized to U6 snRNA expression levels; error bars indicate standard deviation for technical triplicate. Statistically significant difference from U6 control denoted by asterisks (*: two-tailed P-value <0.01).

Many miRNAs expressed in the brain demonstrated dynamic expression changes with age (**Figure 3(a)**). 18 novel miRNA candidates and 75 known miRNAs demonstrated ≥2.0-fold differential expression with age (**Figures 1(a)** **and 3(b)**) (known: 4 upregulated, 71 downregulated; novel: 4 upregulated, 14 downregulated). While specific miRNAs were upregulated in expression with increased age, most of these differentially expressed miRNAs demonstrated decreased expression, in agreement with trends reported in previous studies that profiled miRNA expression changes with age [12], [18]. It is interesting to note that, on average, novel miRNA candidates were detected at copy numbers roughly 3 orders of magnitude lower than that of known miRNAs, providing one explanation as to why they might not have been previously identified. A few known miRNAs were selected for validation by qRT-PCR (**Figure 3(c)**). MiR-124 and miR-34a showed similar expression trends in qPCR and deep sequencing data, but we were not able to detect large changes in expression of miR-17 with age using qPCR. Deep sequencing and qPCR differ on sequence end recognition and are not always comparable, especially in cases where the ends of the miRNA might be modified.

### Targeted Pathways of Age-associated miRNAs

We then asked what biological processes might be affected as a result of differential miRNA expression with age. Because of the differences in expression levels of known and putative novel miRNAs, the two categories of miRNAs were considered separately. We predicted targets for putative novel miRNAs as well as upregulated known miRNAs (predictions were considered for downregulated known miRNAs but not assessed due to the unwieldy number of hits) using a combination of miRanda and TargetScan [23], [24] (see Methods) (**Tables S5 and S6**). Many genes were predicted to be targeted multiple times by a single miRNA, and conversely, many miRNAs were predicted to target a single gene.

**Figure 4:**
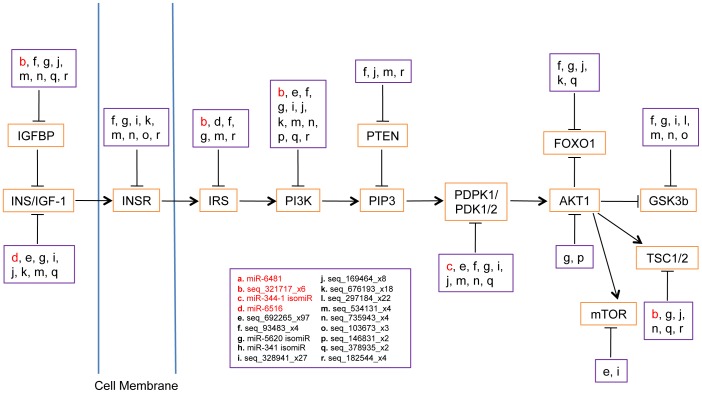
The insulin signaling pathway is predicted to be targeted by many aging-regulated novel miRNA candidates. Multiple novel miRNA candidates are predicted to target each of the genes implicated in the pathway, and each novel miRNA candidate is predicted to target multiple genes in the pathway. In red: upregulated (>2.0-fold) novel miRNAs; in black: downregulated (>2.0-fold) novel miRNAs.

To further elucidate the possible functional relevance of age-affected miRNAs, we next looked for significantly overrepresented KEGG (Kyoto Encyclopedia of Genes and Genomes) pathways (P-value ≤0.001, adjusted for multiple testing (Benjamini & Hochberg method [25])) among the predicted target genes of miRNAs. The KEGG pathway database contains knowledge on molecular interactions in known metabolic and regulatory pathways [26], [27] and allows functional characterization of an input gene list. When targets for individual miRNAs were examined, only a handful of KEGG pathways were found. Examples include endocytosis for seq_321717_x6, an upregulated novel miRNA, and the insulin signaling pathway for seq_735943_x3, a downregulated novel miRNA candidate (**Table S6**). We also examined the collective targets of up- and downregulated novel miRNA candidates as well as upregulated known miRNAs separately, to consider cooperative targeting of pathways by multiple coexpressed miRNAs. We found new significantly targeted KEGG pathways, in addition to those represented by targets of individual miRNAs (**Table S7**). These include the mTOR signaling pathway (P-value = 6.06×10^−10^) and tight junction pathway (P-value = 5.33×10^−10^). Furthermore, pathways targeted by single miRNAs are significantly more enriched when examined as a target of multiple miRNAs. This observation was especially prominent with the insulin signaling pathway (P-value = 1.01×10^−21^). Indeed, many key genes implicated in the insulin signaling pathway are predicted to be targeted by multiple putative novel miRNAs that are downregulated with age (**Figure 4** **and Figure S1**). Figure 4 shows major component genes of the insulin signaling pathway and their predicted targeting status by the novel miRNA candidates in this study.

In order to assess whether differential miRNA expression with age has impacts on putative target gene expression, we next examined mRNA expression data from AGEMAP (Atlas of Gene Expression in Mouse Aging Project) [28]. AGEMAP is a comprehensive analysis of mRNA expression change as a function of time, specifically examining expression changes of 8932 genes over four times points (1, 6, 16, and 24 months) in 16 different mouse tissues including 5 in the central nervous system (cerebrum, cerebellum, hippocampus, striatum, and spinal cord). Looking specifically at tissues within the brain, we retrieved expression data for genes that belong to the insulin signaling pathway and are targeted by novel miRNA candidates downregulated with age reported in this paper. We examined expression at the 6 and 24 month time points from AGEMAP, which match the young and old time points used in our study, respectively. Overall, expression changes in brain tissues were small, but some of the genes examined fell in the top or bottom 5th percentiles of all gene expression changes in each tissue (50th percentile corresponding to fold change ≈1) (**Table S8**, yellow cells). However, the proportion of insulin signaling pathway genes targeted by downregulated novel miRNA candidates that fall within the top or bottom 5th percentiles was not enriched compared to the entire gene set (P-value  = 0.07). Further, only one gene, *Prkar2a*, showed statistically significant expression changes between old and young samples (**Table S8**, green cell). However, of the 43 such insulin signaling pathway genes, 11 were not found in the AGEMAP dataset. These genes are as follows and omitted from **Table S8**: *Gck*, *Insr*, *Pklr*, *Prkaa2*, *Sh2b2*, *Eif4e1b*, *Pik3r5*, *Elk1*, *Ppp1r3a*, *Crkl*, and *Ptpn1*. One of these genes, the insulin receptor (*Insr*), has been shown to increase in protein expression with age [29]. This is in agreement with the predicted reduced targeting of *Insr* expression by novel brain-expressed miRNAs reported in our study.

## Discussion

The role of miRNAs in brain aging is only beginning to be uncovered, and here, we provide evidence that multiple miRNAs, including 41 novel miRNA candidates, may be involved in this complex process. We detected significant differential expression of miRNAs between young and extremely old mouse brains, and many such differentially expressed miRNAs were predicted to target pathways and processes relevant to aging. Interestingly, this aging-associated, pathways-specific targeting was especially prominent among newly discovered miRNAs.

The novel miRNA candidates we report in this study may represent a class of aging-specific miRNAs expressed only in restricted regions of the brain. As our RNA samples were derived from whole brains of mice, it is not possible to determine which specific, possibly aging-relevant regions of the brain these novel miRNAs were expressed. Still, the fact that most novel miRNAs were expressed at levels much lower than that of known miRNAs is suggestive of region-specific expression. Further, nearly 15% of the brain-expressed miRNAs we report in this study are novel. Brain-expressed miRNAs have been investigated extensively, yet a recently conducted deep sequencing study of the developing mouse brain found only 4 novel miRNAs [30]. The context-specificity of the expression of some miRNAs is well known, and it is possible that the novel miRNAs we report in this study are expressed only under the specific circumstances associated with aging. Future studies could use *in situ* hybridization to address region- and context-specificity.

Additional evidence suggesting that these novel miRNA candidates are relevant to aging regulation comes from their predicted targets. Individual miRNAs and multiple miRNAs collectively were predicted to target such aging-relevant pathways as the insulin signaling pathway. The insulin signaling pathway plays a central, conserved role in lifespan determination, with many genes affecting lifespan when mutated [31]. In mice, animals that are mutant for the insulin/insulin-like growth factor 1 receptor (IGF-1R) exhibit long life and stress resistance [32]. Additionally, increases in brain IGF-1R and compromised insulin receptor signaling have been associated with neurodegenerative diseases such as Alzheimer’s disease [33]. The novel miRNAs we report here may contribute to both normal and pathological aging through their potential functions in regulating the insulin signaling pathway.

AGEMAP mRNA expression data did not show large changes in expression of putative novel miRNA-targeted insulin signaling pathway genes with age. However, 11 of the 43 insulin signaling pathway genes predicted to be targeted by downregulated novel miRNAs were not included in the AGEMAP analysis. One of these genes was *Insr*. Increased brain INSR expression with age has been shown by Muller *et al*. [29], and this is in agreement with the age-related reduction of novel miRNAs predicted to target *Insr* expression. We also looked at a list of genes showing increased expression with age in the neocortex generated by Lee *et al.* comparing mice at 5 and 30 months of age [6] and found that one gene, *Akt2*, was also predicted to be targeted by downregulated novel miRNAs we report here (**Table S8**, red cell). That miRNAs exhibit dynamic expression changes with age while mRNAs do not could be suggestive of a role for miRNAs in buffering molecular changes during aging. Moreover, miRNAs function by targeting multiple genes and working in cooperation with other miRNAs; and we do not fully understand how much individual miRNAs contribute to each mRNA’s expression. Further research is needed to elucidate the roles of miRNAs, individually and collectively, in aging.

Many of the miRNAs that we detect in our study have been identified by others as brain-expressed miRNAs, thus validating our method for its ability to detect brain-expressed miRNAs. For example, miR-124 was found by Lagos-Quintana and colleagues as dominating the population of brain-expressed miRNAs in young mice [14]. While not the predominating miRNA, miR-124 is strongly represented in our set of aging brain-expressed miRNAs. Other brain-specific miRNAs [14] included in our dataset include miR-101, -127, -128, and -132.

Among the most abundantly expressed miRNAs in our dataset were the *let-7* family miRNAs. *let-7* family members have been noted for their high representation in the mouse as well as primate brains [14], [15]. Further, these miRNAs generally demonstrated increased expression with age in our dataset (**Table S1 and S2**). This trend is in agreement with the results of a recent study which used size-coded ligation-mediated PCR and found increased *let-7* family miRNA expression levels in the aged mouse brain [34]. It is interesting to note that increased *let-7b* expression has been reported in aged mouse neural stem cells and maybe contributing to decreased stem cell function by targeting *Hmga2* expression [35]. While *let-7b* expression was not significantly altered during brain aging in our dataset, it is possible that other *let-7* family members, like *let-7f* which is increased more than 2-fold with age and shares identical seed sequences with *let-7b*, could play regulatory roles in brain aging.

Some of the aging brain-associated miRNAs have been implicated in neurodegenerative diseases. For example, miR-101 and miR-433, both significantly downregulated in our dataset, have been linked to spinocerebellar ataxia type 1 (SCA1) and Parkinson’s disease, respectively [36], [37]. miR-101 targets ataxin 1, responsible for SCA1, at both the mRNA and protein levels; miR-433 targets fibroblast growth factor 20 which induces α-synuclein expression, which was previously shown to cause Parkinson’s disease when overexpressed [38]. The inclusion of miRNAs known to be relevant to neurodegenerative disease pathogenesis in our dataset suggests that at least some gene expression changes in normal and pathological aging are common, substantiating the need for a better and deeper understanding of the mechanisms that distinguish them.

The majority of our aging brain-expressed miRNAs were also detected in a recent study that profiled the developing mouse brain [30]. Many studies have linked development and aging [39], [40], and the first miRNA discovered to be implicated in lifespan regulation, *lin-4*, functions in the regulation of developmental timing [11]. The overlapping miRNA repertoire during development and aging suggests a direct link between developmental regulation and aging. Somel and colleagues found that some miRNA–target relationships in development and aging in humans may be correlated, implicating that at least some expression changes that take place during aging are driven by the same mechanism as in development [41].

Some annotated miRNAs that are known to target genes in the insulin signaling pathway were also affected in our dataset. MiR-145, which was shown to target *Irs-1* and *Irs-2* [42], was downregulated with age. MiR-375 targets *Pdk-1* and decreases downstream insulin signaling [43]; this miRNA is among the most downregulated miRNAs with age in our dataset. These findings support the involvement of aging-affected miRNAs in aging-associated pathways.

Lastly, we compared our list of miRNAs most changed in expression levels during brain aging to those shown to change their expression in other aging tissues. Wang and colleagues have published microarray studies on miRNAs in the mouse liver and brain [44], [45]. Contrary to our and others’ findings that miRNA expression is generally decreased with age in varying tissues and organisms (this work and [12], [18]), Wang and colleagues report predominant miRNA upregulation in aging tissues [44], [45]. Comparing our brain study with theirs, it is important to note that different time points, brain tissues (whole vs. exclusion of cerebellum in Li *et al.* [45]), and methods were employed, providing possible explanations for the different conclusions. The fact that many of the miRNAs they report as changing with age are also found in our dataset lends support to the importance of those miRNAs in the aging process.

The full scope of miRNA functions has yet to be elucidated. The differential expression of many miRNAs during aging supports their wide involvement in yet another complex process that is not well understood molecularly. The pattern of altered miRNA expression we observe here suggests that multiple gene regulatory relationships are affected in aging and in particular that the highly conserved insulin signaling pathway maybe strongly affected. These findings point to novel functions for miRNAs in the molecular mechanisms of aging. The highly conserved nature of miRNAs between mice and humans promises that findings from this study will be relevant and applicable to human gerontology research.

## Materials and Methods

### Animals

Male mice (C57BL/6J strain), two aged 5 months (young batch) and two aged 24–25 months (old batch), were chosen using the following criteria: appearance of health, age (as old as possible, for old batch), and sexual maturity (applicable to young mice only). Mice were sacrificed and brain tissues were immediately removed and flash frozen in liquid nitrogen. Before freezing, tissues were analyzed histologically and found to be healthy. Mice were also analyzed histologically by dissection and found to be in relatively good condition, without visible signs of tumorigenesis or disease (data not shown). All animal experiments were performed under an animal study protocol approved by Yale University’s Institutional Animal Care and Use Committee.

### RNA Preparation

RNA from mouse brains was extracted as previously described [46]. In short, frozen tissues were homogenized, and lysates were thawed and phenol extracted. Resulting RNA pellets were dissolved in Nuclease Free water (Ambion, Austin, TX, USA), and stored at −80°C.

### Small RNA Isolation and Deep Sequencing

Small RNAs were isolated and cloned as described previously [47]. Briefly, small RNAs from young and old mouse brains were purified and prepared for Illumina sequencing, according to the manufacturer’s recommendations. 10 µg of total RNA extracted from each of four mouse brains (two old and two young) were electrophoresed through a 15% TBE-urea PAGE gel. The fraction of the lanes containing RNA molecules between 18–30 nucleotides in length was excised from the gel. RNA was isolated from the gel slices and ethanol precipitated. Isolated small RNAs were then fitted with 5′ and 3′ adapters, and reverse transcribed, all in accordance with the manufacturer’s protocol. Resulting cDNA was PCR amplified using Illumina primers, and PCR amplicons were electrophoresed, excised from polyacrylamide gels and ethanol precipitated. Purified DNA libraries from the olds brains were combined, as were purified DNA libraries from the young brains. Each cDNA library (old mix and young mix) was subsequently sequenced by Illumina sequencing technology. A total of 14,034,295 raw sequences of small RNAs were obtained by Solexa deep sequencing (Illumina) (read length: 36 nt). The software program SOAP [48] was used to align raw sequences to the reference mouse genome, mm9, NCBI Build 37, (obtained from http://genome.ucsc.edu) and sequences corresponding to the 3'-end cloning adapter TCGTATGCCGTCTTCTGCTTGT were removed, allowing a maximum of 3 mismatches in the adapter (option -S 3 in Soap). The number of sequence reads that correspond to known miRNAs was determined by perfect sequence matching to the database of known miRNAs (miRBase release 16). The R package DEGseq was used to identify differentially expressed miRNAs [20]. P-values for differentially expressed miRNAs were calculated in DEGseq using the MA-plot-based random sampling model (MARS). Raw sequencing reads have been deposited in the NCBI Gene Expression Omnibus with the accession number GSE34393.

### Novel miRNA Discovery

We used the software miRDeep [21] to identify novel miRNAs in our deep sequencing data. Beginning with 11,181,999 genome-matching reads, we discarded sequences that correspond to previously annotated, non-coding RNAs and mRNAs using scripts in the miRDeep package after identifying matches in our dataset using NCBI megablast (version 2.2.17). The sequences of previously identified non-coding RNAs and mRNAs were obtained from the fRNAdb non-coding RNA sequence database (downloaded on 2009/10/21 from http://www.ncrna.org/frnadb) and UCSC genome browser (mrna.fa.gz from mm9 mouse genome), respectively. In addition, matches to known *Mus musculus* miRNAs (miRBase release 16) were removed by perfect sequence matching. No Piwi-interacting RNAs (piRNAs) were identified in our brain sequencing data (consistent with their specific expression in mouse testis [49]). The remaining, non-annotated reads were then mined for characteristics of novel miRNAs using miRDeep.pl, using default settings [21]: these characteristics include the presence of reads corresponding to typical products of miRNA biogenesis; stability of the putative pre-miRNA hairpin; and homology to previously identified miRNAs. We considered miRNAs conserved if at least 50% of the overall sequence is identical and all of the seed sequence (nucleotides 2–7/8) matched separately. Results were individually confirmed by BLAST.

### MiRNA Target Prediction and Functional Analysis

Targets for known and novel miRNAs were predicted using miRanda and TargetScan 5.0 software [23], [24] against 3′ UTR sequences obtained from the Ensembl and UCSC databases. Four separate predictions were conducted with different combinations of prediction software and 3′ UTR sets (TS5.0 with Ensembl-derived 3′ UTR, miRanda with Ensembl-derived 3′ UTR, TS5.0 with UCSC-derived 3′ UTR, and miRanda with UCSC-derived 3′ UTR). Only common predictions were considered. Because TargetScan and miRanda predicted different numbers of target sites, the more conservative number was taken as the reported number of predicted target sites for a given miRNA–mRNA pair; these numbers were later used to rank target predictions. Gene annotations were retrieved using BioMart [50], [51]. KEGG pathways were retrieved using WebGestalt [52], [53] for genes with more than 2 predicted miRNA target sites. P-value cut-off <0.001 was chosen based on the recommendations in [54] for the application of a higher significance threshold for robust functional profiling of miRNA targets. P-values were corrected for multiple-hypothesis testing as part of the WebGestalt analysis using the Benjamini & Hochberg method [25]. MiRNA sequence alignment was conducted using Clustal W 2.0 [55], [56].

Microarray expression profiling data of the following tissues in the brain of aging mice were retrieved from AGEMAP [28]: cerebrum, cerebellum, hippocampus, and striatum. Mice used in AGEMAP were C57BL/6 strain; in our study, we used C57BL/6J strain mice, a subline of C57BL/6. In order to compare with our dataset, only data for male mice aged 6- and 24-months old were analyzed; these time points correspond closely to the ages of mice used in our experiment. Percentile rank of expression change was calculated relative to the expression change of all 8932 genes assessed in each tissue. Statistical significance was determined by Welch’s two sample t-test (P-value <0.05). Of the 43 insulin signaling pathway genes predicted to be targeted by downregulated novel miRNAs, 11 were not found in the AGEMAP dataset. These genes are: *Gck*, *Insr*, *Pklr*, *Prkaa2*, *Sh2b2*, *Eif4e1b*, *Pik3r5*, *Elk1*, *Ppp1r3a*, *Crkl*, and *Ptpn1*.

### Quantitative Reverse Transcription PCR

TaqMan MicroRNA Assays (Applied Biosystems, Foster City, CA, USA) were used according to manufacturer’s recommendations. MiRNA-specific primers for reverse transcription and qPCR were designed for nine novel miRNAs (all four upregulated miRNAs and top five downregulated miRNAs) (**Figure 1(a)**). Probes for snRNA U6, miR-124 and miR-155 were used as controls for the qRT-PCR. Expression levels relative to that of U6 were determined using the 2^-ΔΔC^_T_ method [57]. Statistical significance of expression change was determined by unpaired t-test.

## Supporting Information

Figure S1 **Insulin signaling pathway genes that are predicted to be targeted by novel miRNAs.** Pathway retrieved from KEGG [26], [27]. Boxes highlighted in red indicate genes that are predicted to be targeted by at least one novel miRNA.
(PDF)

Table S1 **All 558 known miRNAs expressed in the mouse brain.**
(XLSX)

Table S2 **All known miRNAs expressed in the mouse brain that are differentially expressed (P-values <0.05).** Red: greater than 2.0-fold increase in old versus young mouse brains. Green: greater than 2.0-fold decrease in old versus young mouse brains. MiRNAs with read counts ≥10 in at least one time point sample indicated in bold.
(XLSX)

Table S3 **MiRNA star sequences expressed in the mouse brain.** Red: greater than 2.0-fold increase in old versus young mouse brains. Green: greater than 2.0-fold decrease in old versus young mouse brains. Differentially expressed miRNAs with P-value <0.05 indicated in bold.
(XLSX)

Table S4 **Novel miRNA candidates expressed in the mouse brain.** MiRNA frequency was normalized by all reads that matched to the mouse genome (mm9) (Old/Young  = 1.472107). †: candidates tested for by qRT-PCR. ‡: candidates with sequence overlap with known miRNAs (isomiRs). Blue font: miRNA new to mouse. Black font: completely novel miRNA sequence. * P-values for significance of differential expression calculated using DEGseq [20]. P-values <0.05 indicated in bold. Three novel miRNA candidates overlap with snoRNA sequences (miR-6516, seq_692265_x97, seq_93483_x4) Two candidates mapped to multiple genomic regions including rRNA sequences when BLASTed. age: *Ateles geoffroyi*. bta: *Bos taurus*. dan: *Drosophila ananassae*. der: *Drosophila erecta*. dgr: *Drosophila grimshawi*. dme: *Drosophila melanogaster*. dmo: *Drosophila mojavensis*. dpe: *Drosophila persimilis*. dps: *Drosophila pseudoobscura*. dse: *Drosophila sechellia*. dsi: *Drosophila simulans*. dvi: *Drosophila virilis*. dwi: *Drosophila willistoni*. dya: *Drosophila yakuba*. gga: *Gallus gallus*. ggo: *Gorilla gorilla*. hsa: *Homo sapiens*. lla: *Lagothrix lagotricha*. mdo: *Monodelphis domestica*. mml: *Macaca mulatta*. mne: *Macaca nemestrina*. oan: *Ornithorhynchus anatinus*. ppa: *Pan paniscus*. ppy: *Pongo pygmaeus*. ptr: *Pan troglodytes*. sla: *Saguinus labiatus*. sme: *Schmidtea mediterranea*. xtr: *Xenopus tropicalis*.
(XLSX)

Table S5 **Genes targeted by upregulated known miRNAs (≥2 target sites) and KEGG pathways (P-value >0.001) represented by those target genes.**
(XLSX)

Table S6 **Genes and pathways targeted by up- and downregulated novel miRNA candidates.** **(a)** Genes targeted by upregulated novel miRNA candidates (≥2 target sites) and KEGG pathways represented by those target genes (P-value >0.001). **(b)** Genes targeted by downregulated novel miRNA candidates (≥2 target sites) and KEGG pathways represented by those target genes (P-value >0.001).
(XLSX)

Table S7 **KEGG pathways targeted by multiple novel miRNA candidates (upregulated novel miRNAs or downregulated novel miRNAs, collectively).** (P-value >0.001).
(XLSX)

Table S8 **Expression data of insulin signaling pathway genes retrieved from AGEMAP** [**28**] **.** Expression levels of insulin signaling genes in four tissues of the brain were compared for 24 month- vs. 6 month-old mice samples. Percentile ranks of these expression fold changes relative to expression changes of all 8932 genes assayed in each tissue are shown. **(a)** Genes predicted to be targeted by seq_735943_x3. **(b)** Genes predicted to be targeted by 14 novel downregulated miRNAs. Yellow: genes demonstrating expression fold change falling in the top or bottom 5th percentile of all gene expression changes. Green: gene demonstrating significant expression change in 24 month- vs. 6 month-old animals (P-value <0.05, Welch’s two sample t-test). Red: gene also found in Lee *et al.* [6] as demonstrating increased expression with age in the neocortex (1.8-fold increase in 30- vs. 5-month old mice). Of the 43 insulin signaling pathway genes predicted to be targeted by downregulated novel miRNAs, 11 were not found in the AGEMAP dataset. These genes are: *Gck*, *Insr*, *Pklr*, *Prkaa2*, *Sh2b2*, *Eif4e1b*, *Pik3r5*, *Elk1*, *Ppp1r3a*, *Crkl*, and *Ptpn1*.
(XLSX)
