# Supplementary figures and images for: Novel MicroRNAs Differentially Expressed during Aging in the Mouse Brain
Source: PLoS One. 2012 Jul 23;7(7):e40028. doi: 10.1371/journal.pone.0040028 (PMC3402511; doi:10.1371/journal.pone.0040028)

Figure S1

INSULIN SIGNALING PATHWAY

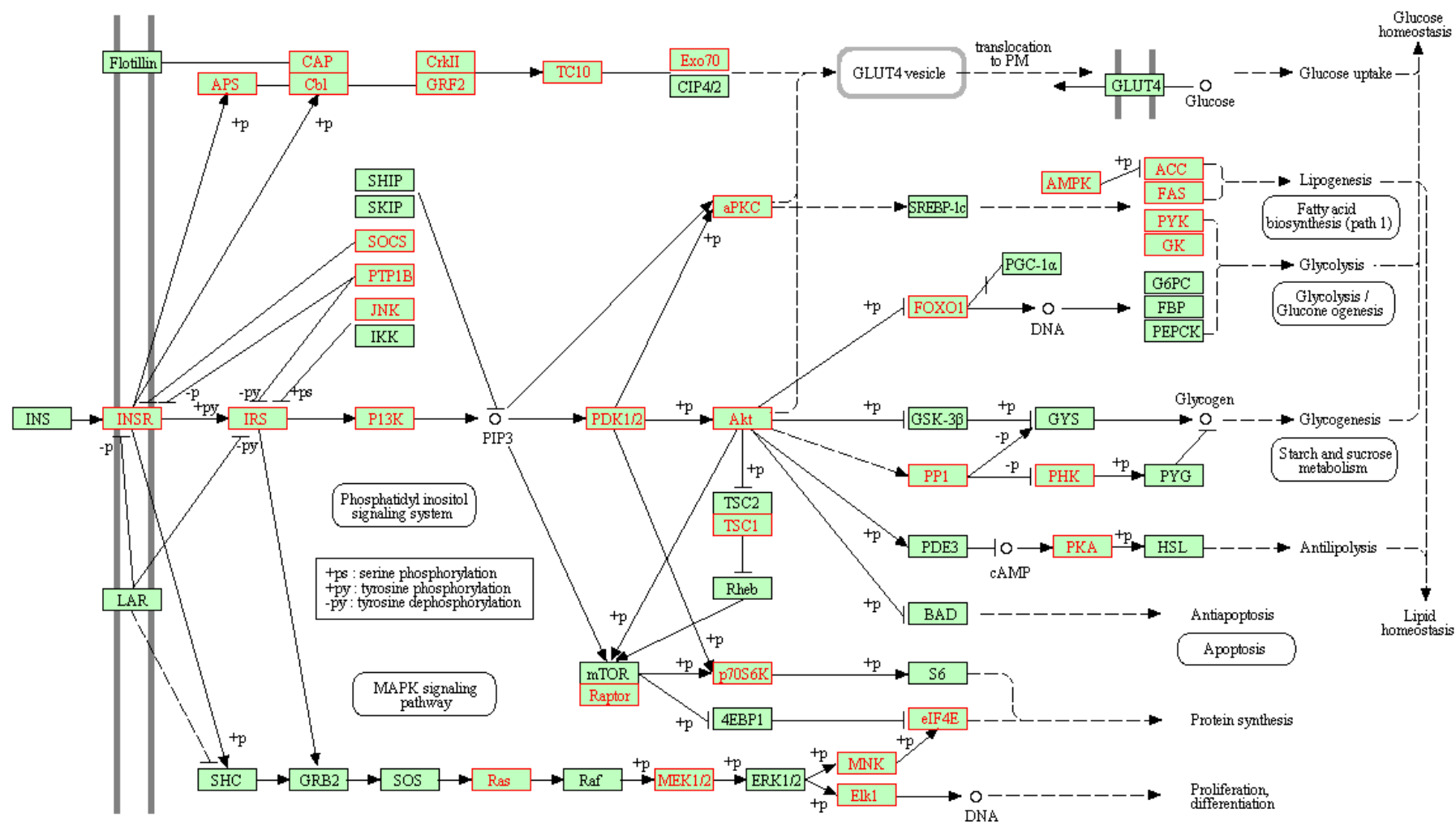

Supplement: Figure S1 — Insulin signaling pathway genes that are predicted to be targeted by novel miRNAs. Pathway retrieved from KEGG [26], [27]. Boxes highlighted in red indicate genes that are predicted to be targeted by at least one novel miRNA. (PDF) [file pone.0040028.s001.pdf]
